# Supplementary material for: BactMentha: a database of annotated bacteria-host protein interactions
Source: Database (Oxford). 2026 Nov 14;2026:baag068. doi: 10.1093/database/baag068 (PMC13768950; doi:10.1093/database/baag068)
Supplement: Supplementary file 1 [file baag068_supplemental_file.docx]

## Supplementary Data

**BactMentha: a database of annotated bacteria-host protein interactions**

Lou Bergogne^1^, Mégane Boujeant^1^, Marta Iannuccelli^2^, Aurélie Bergon^1^, Jaime Fernandez-Macgregor^1,3^, Christine Brun^1,4^, Luana Licata^2^, Andreas Zanzoni^1, #^

1 Aix Marseille University, INSERM, TAGC, UMR 1090, Marseille, France. 2 Department of Biology, University of Rome Tor Vergata, Rome 00133, Italy. 3 Aix-Marseille University, CNRS, AFMB, UMR CNRS 7257, Turing Center for Living Systems, Marseille, France. 4 CNRS, Marseille, France

\# To whom correspondence should be addressed: andreas.zanzoni@univ-amu.fr.

**SUPPLEMENTARY FIGURES**

**Supplementary Figure 1. Overlap among predicted and experimentally identified binding regions.** BactMentha contains experimentally identified binding regions in at least one interaction partner for 532 bacteria-host interactions. For a subset of bacteria-human protein interactions, it also provides putative molecular interfaces predicted with *mimic*INT (1043 protein interactions) and AlphaFold3 (379 protein interactions). One-hundred fifty-six (156) interactions have at least one experimentally identified and one predicted interface. In total there are 1733 protein interactions for which predicted and/or experimentally identified molecular binding regions are available.

**Supplementary Figure 2. BactMentha website Advanced Search page.** Accessible from the Home page, the Advanced Search page provides a user guide defining the specificities of the advanced search queries such as authorized formats (panel 1), the different conditions or the way the distinct conditions will be joined in the resulting query to the database, along with some examples of use on the right side. The advanced search form contains constructed conditions joined by AND or OR clauses (panel 2): a feature associated to a condition (is equal to, is different forms, contains or doesn’t contain) and some comparison element typed in the following text field following the rules displayed in the user guide. New conditions can be added with the *‘Add condition’* button or some can be removed using the bin button. The form can be cleared using the *‘Reset’* button or the query can be launched by clicking the *‘Search’* button.

**Supplementary Figure 3. BactMentha browse page.** The Browse page provides direct access to specific bacteria-host datasets as well as pre-complied data views based on WHO priority list and HSE Hazard groups. Some pre-filtered tables shortcuts are also available to display the bacteria-human protein interactions for which experimental binding regions or predicted interfaces are available.

**Supplementary Figure 4. BactMentha download page.** The Download page provides the user the archive files of the current (latest tag) and previous version of the BactMentha database. The user can download a complete version of the database, the formatted database tables (including annotations tables such as experimental binding regions, *mimic*INT and AF3 interfaces and bacterial protein annotations) or the reference data (derived from each step of the BactMentha workflow), either for all or individual hosts.

**Supplementary Figure 5. The CBU0175-RBPMS predicted complex.** AF3 predicted complex between the Coxiella *burnetii* effector protein CBU0175 (UniProt identifier: Q820B6) and the human RNA-binding protein with multiple splicing (RBPMS, UniProt identifier: Q93062) with an ipTM=0.64. AF3 predicted the interface between the serine/threonine protein kinase domain of CBU0175 and the RRM (RNA recognition motif) domain of RBPMS. The figure was generated using ChimeraX (version 1.10, <https://www.cgl.ucsf.edu/chimerax/>).

**SUPPLEMENTARY TABLES**

**Supplementary Table 1. List of the articles specifically curated for BactMentha**. For each article the following information is reported: PubMed identifier, digital object identifier (DOI) if available (NA otherwise), journal title abbreviation and publication year.

| **PubMed Identifier** | **DOI** | **Journal** | **Year** |
| --- | --- | --- | --- |
| 11093251 | NA | Cell Motil Cytoskeleton | 2000 |
| 12446738 | NA | J Biol Chem | 2003 |
| 15527496 | NA | Cell Microbiol | 2004 |
| 15296718 | NA | Dev Cell | 2004 |
| 14710194 | NA | EMBO Rep | 2004 |
| 16480265 | NA | J Med Chem | 2006 |
| 16367866 | NA | Cell Microbiol | 2006 |
| 16354697 | NA | Mol Cell Biol | 2006 |
| 18060821 | NA | Mol Cell | 2007 |
| 17451412 | NA | Cell Microbiol | 2007 |
| 17405911 | NA | Ann N Y Acad Sci | 2007 |
| 17237808 | NA | Oncogene | 2007 |
| 17160020 | NA | Oncogene | 2007 |
| 18974829 | 10.1371/journal.ppat.1000191 | PLoS Pathog | 2008 |
| 17979986 | NA | Cell Microbiol | 2008 |
| 19604117 | 10.1086/604727 | J Infect Dis | 2009 |
| 19273555 | 10.1128/IAI.00027-09 | Infect Immun | 2009 |
| 21187904 | 10.1371/journal.ppat.1001231 | PLoS Pathog | 2010 |
| 20618342 | 10.1111/j.1462-5822.2010.01503.x | Cell Microbiol | 2010 |
| 20585566 | 10.1371/journal.ppat.1000960 | PLoS Pathog | 2010 |
| 21911467 | 10.1128/IAI.05622-11 | Infect Immun | 2011 |
| 21841197 | 10.1074/jbc.M111.263855 | J Biol Chem | 2011 |
| 21569203 | 10.1111/j.1742-4658.2011.08159.x | FEBS J | 2011 |
| 21525010 | 10.1074/jbc.M110.184283 | J Biol Chem | 2011 |
| 21148319 | 10.1074/jbc.M110.172254 | J Biol Chem | 2011 |
| 22587461 | 10.1042/BJ20120533 | Biochem J | 2012 |
| 22544751 | 10.1074/jbc.M112.363473 | J Biol Chem | 2012 |
| 23797873 | 10.1038/embor.2013.86 | EMBO Rep | 2013 |
| 23516580 | 10.1371/journal.pone.0058937 | PLoS One | 2013 |
| 23474333 | 10.1016/j.micinf.2013.02.004 | Microbes Infect | 2013 |
| 23434013 | 10.1016/j.vetmic.2013.01.028 | Vet Microbiol | 2013 |
| 25513777 | 10.4161/viru.29062 | Virulence | 2014 |
| 25193659 | 10.1074/jbc.M114.604876 | J Biol Chem | 2014 |
| 25114243 | 10.1073/pnas.1405391111 | Proc Natl Acad Sci U S A | 2014 |
| 24902122 | 10.1128/mBio.01178-14 | mBio | 2014 |
| 24583011 | 10.1016/j.febslet.2014.02.034 | FEBS Lett | 2014 |
| 26602083 | 10.1016/j.febslet.2015.11.017 | FEBS Lett | 2015 |
| 26419332 | 10.1038/srep14602 | Sci Rep | 2015 |
| 26118995 | 10.1016/j.chom.2015.06.004 | Cell Host Microbe | 2015 |
| 25944859 | 10.1128/mBio.00354-15 | mBio | 2015 |
| 25944661 | 10.1016/j.bbrc.2015.04.116 | Biochem Biophys Res Commun | 2015 |
| 26714771 | 10.1186/s12864-015-2351-1 | BMC Genomics | 2015 |
| 27442026 | 10.1021/acs.biochem.6b00124 | Biochemistry | 2016 |
| 29176882 | 10.1371/journal.pone.0188071 | PLoS One | 2017 |
| 28656718 | 10.1002/1873-3468.12734 | FEBS Lett | 2017 |
| 28602400 | 10.1016/j.micres.2017.04.008 | Microbiol Res | 2017 |
| 28258151 | 10.1042/BCJ20170085 | Biochem J | 2017 |
| 30571761 | 10.1371/journal.ppat.1007491 | PLoS Pathog | 2018 |
| 30323948 | 10.1038/s41421-018-0055-9 | Cell Discov | 2018 |
| 30279169 | 10.1126/scisignal.aat8335 | Sci Signal | 2018 |
| 30212651 | 10.1016/j.chom.2018.08.004 | Cell Host Microbe | 2018 |
| 30118682 | 10.1016/j.molcel.2018.07.010 | Mol Cell | 2018 |
| 29734393 | 10.1371/journal.ppat.1007023 | PLoS Pathog | 2018 |
| 29433439 | 10.1186/s12866-017-1147-7 | BMC Microbiol | 2018 |
| 29328525 | 10.1111/1348-0421.12573 | Microbiol Immunol | 2018 |
| 29165646 | 10.1093/nar/gkx1173 | Nucleic Acids Res | 2018 |
| 29106533 | 10.1093/femsle/fnx231 | FEMS Microbiol Lett | 2018 |
| 31611645 | 10.1038/s41564-019-0580-9 | Nat Microbiol | 2019 |
| 31231326 | 10.3389/fmicb.2019.01218 | Front Microbiol | 2019 |
| 31162724 | 10.1002/prot.25754 | Proteins | 2019 |
| 30962357 | 10.1128/JB.00013-19 | J Bacteriol | 2019 |
| 30909843 | 10.1080/15548627.2019.1590519 | Autophagy | 2019 |
| 30902834 | 10.1074/mcp.RA118.001093 | Mol Cell Proteomics | 2019 |
| 30827827 | 10.1016/j.chom.2019.01.006 | Cell Host Microbe | 2019 |
| 30610939 | 10.1016/j.neuroscience.2018.12.045 | Neuroscience | 2019 |
| 31992623 | 10.1128/mBio.03141-19 | mBio | 2020 |
| 34237247 | 10.1016/j.chom.2021.06.004 | Cell Host Microbe | 2021 |
| 32116095 | 10.1080/15548627.2020.1728098 | Autophagy | 2021 |
| 35963849 | 10.1038/s41420-022-01134-x | Cell Death Discov | 2022 |

**Supplementary Table 2**. Column definitions of the PSI-MITAB27 formatted interaction file fetched via the PSICQUIC web service. The columns stored in the BactMentha database are indicated. Definitions were taken from <https://code.google.com/archive/p/psimi/wikis/PsimiTab27Format.wiki>.

| **Column number** | **Column definition** | **In BactMentha** |
| --- | --- | --- |
| 1 | **Unique identifier for interactor A**  Represented as databaseName:identifier, where databaseName is the name of the corresponding database and identifier is the unique primary identifier of the molecule in the database.  *Ex: uniprotkb:P12346* | **Yes** |
| 2 | **Unique identifier for interactor B** | **Yes** |
| 3 | **Alternative identifier for interactor A**. | Not considered |
| 4 | **Alternative identifier for interactor B**. | Not considered |
| 5 | **Aliases for A**  Separated by "\|". Representation as databaseName:name(alias type), where databaseName is the name of the corresponding database, name is the alias name and alias type is the name of the corresponding alias type.  *Ex: uniprotkb:Tf(gene name)\|uniprotkb:Serotransferrin(recommended name)\|uniprotkb:Tf(display_short)* | **Yes** |
| 6 | **Aliases for B** | **Yes** |
| 7 | **Interaction detection methods**  Represented as databaseName:identifier(methodName), separated by "\|". As the detection methods are taken from the PSI-MI ontology, the database name is 'psi-mi'.  *Ex: psi-mi:"MI:0006"(anti bait coimmunoprecipitation)* | Yes |
| 8 | **First author** | Not considered |
| 9 | **Identifier of the publication**  Represented as databaseName:identifier. Multiple identifiers separated by "\|".  *Ex: pubmed:16980971\|imex:IM-1* | **Yes** |
| 10 | **NCBI Taxonomy identifier for interactor A**  Represented as taxid:identifier(organism name) where the identifier is the taxon id of the organism and organism name can either be the common name or scientific name. If both scientific name and common name are given, they should be represented with : *taxid:id1(common name1)\|taxid:id1(scientific name1)*.  Note: Currently no taxonomy identifiers other than NCBI taxid are anticipated. | **Yes** |
| 11 | **NCBI Taxonomy identifier for interactor B** | **Yes** |
| 12 | **Interaction types**  Represented as dataBaseName:identifier(interactionType), separated by "\|". As the interaction types are taken from the PSI-MI ontology, the database name is 'psi-mi'.  *Ex: psi-mi:"MI:0914"(association)* | **Yes** |
| 13 | **Source databases** | Not considered |
| 14 | **Interaction identifier(s)** | Not considered |
| 15 | **Confidence score**  Denoted as scoreType:value. Multiple scores separated by "\|". *Ex: author-score:0.60\|author-score:high\|intact-miscore:0.36784992* | **Yes** |
| 16 | **Complex expansion** | Not considered |
| 17 | **Biological role A** | Not considered |
| 18 | **Biological role B** | Not considered |
| 19 | **Experimental role A** | Not considered |
| 20 | **Experimental role B** | Not considered |
| 21 | **Interactor type A** | Not considered |
| 22 | **Interactor type B** | Not considered |
| 23 | **Xref for interactor A** | Not considered |
| 24 | **Xref for interactor B** | Not considered |
| 25 | **Xref for the interaction** | Not considered |
| 26 | **Annotations for interactor A** | Not considered |
| 27 | **Annotations for Interactor B** | Not considered |
| 28 | **Annotations for the interaction** | Not considered |
| 29 | **NCBI Taxonomy identifier for the host organism** | Not considered |
| 30 | **Parameters of the interaction** | Not considered |
| 31 | **Creation date** | Not considered |
| 32 | **Update date** | Not considered |
| 33 | **Checksum for interactor A** | Not considered |
| 34 | **Checksum for interactor B** | Not considered |
| 35 | **Checksum for interaction** | Not considered |
| 36 | **negative** | Not considered |
| 37 | **Feature(s) for interactor A**  Describe features for participant A such as binding sites, PTMs, tags, etc. Represented as feature_type:range(text). For instance : sufficient to bind:27-195,201-133 (IPR000785). The use of the following characters is allowed to describe a range position : ‘?’ (undetermined position), ‘n’ (n terminal range), ‘c’ (c-terminal range), ‘>x’ (greater than x), ‘<’ (less than x), ‘x1..x1’ (fuzzy range position *Ex : 5..5-9..10*). The character '-' is used to separate start position(s) from end position(s). Multiple features separated by '\|'. Multiple ranges per feature separated by ','.  *Ex: gst tag:n-n(n-terminal region)\|sufficient to bind:23-45. or binding site:23..24-46,33-33* | **Yes** |
| 38 | **Feature(s) for interactor B** | **Yes** |
| 39 | **Stoichiometry for interactor A** | Not considered |
| 40 | **Stoichiometry for interactor B** | Not considered |
| 41 | **Participant identification method for interactor A** | Not considered |
| 42 | **Participant identification method for interactor B** | Not considered |

**Supplementary Table 3.** Summary of the AF3 complex predictions. We prioritized a subset of interaction by selecting clinically relevant strains (i.e., hazard group 2 or 3) encompassing different infection strategies (i.e., extracellular/adhesive or intracellular) and secretory system types (e.g., T3 or T4). We have predicted structural models for 379 interactions between 60 annotated bacterial and 337 human proteins. Failed predictions for 7 interactions were mainly due to missing amino acids in bacterial sequences and excessive length of human protein sequences.

| **Strain name (taxon id)** | **Hazard group** | **# AF3 predicted complexes (n=379)** | **# failed A3F predictions (n=7)** |
| --- | --- | --- | --- |
| **Escherichia coli O111:H- (585396)** | 3 | 1 | 0 |
| **Escherichia coli O127:H6, EPEC (574521)** | 2 | 33 | 0 |
| **Escherichia coli O157:H7 (83334)** | 3 | 79 | 5 |
| **Mycobacterium tuberculosis, H37Rv (83332)** | 3 | 12 | 0 |
| **Mycobacterium tuberculosis, Erdman (652616)** | 3 | 24 | 1 |
| **Coxiella burnetii, RSA 493 (227377)** | 3 | 230 | 1 |

**Supplementary Table 4.** Functional annotations of bacterial proteins in BactMentha based on BastionHub and VFDB databases. This table provides an overview of the functional categories assigned to bacterial proteins in BactMentha after BLASTp sequence comparison against the protein sequences contained in BastionHub and Virulence Factor Database. It includes the number of proteins annotated to each category, the source of the annotation, and the number of PPIs involving bacterial proteins for each functional annotation.

| **Annotation Description** | **Annotated proteins** | **Annotation source** | **interactions** |
| --- | --- | --- | --- |
| **Type 3 secretion system** | 136 | BastionHub | 2152 |
| **Type 4 secretion system** | 26 | BastionHub | 159 |
| **Type 6 secretion system** | 5 | BastionHub | 18 |
| **Type 1 secretion system** | 4 | BastionHub | 14 |
| **Effector delivery system** | 325 | VFDB | 3336 |
| **Nutritional/Metabolic factor** | 105 | VFDB | 307 |
| **Adherence** | 77 | VFDB | 248 |
| **Motility** | 62 | VFDB | 216 |
| **Stress survival** | 32 | VFDB | 189 |
| **Exotoxin** | 57 | VFDB | 159 |
| **Immune modulation** | 62 | VFDB | 157 |
| **Biofilm** | 19 | VFDB | 71 |
| **Exoenzyme** | 14 | VFDB | 60 |
| **Invasion** | 11 | VFDB | 44 |
| **Regulation** | 16 | VFDB | 34 |
| **Others** | 16 | VFDB | 28 |
| **Antimicrobial activity/Competitive advantage** | 8 | VFDB | 18 |
| **Post-translational modification** | 1 | VFDB | 5 |

**Supplementary Table 5.** *mimic*INT predicted interfaces for the interactions between *Coxiella burnetii* and human proteins. ProtA: identifier of the bacterial protein; ProtA_interface_id: Identifier of the predicted interface (either an InterPro domain or a ELM motif); ProtA_interface_start: starting amino acid position of the predicted interface on Protein A; ProtA_interface_end: ending amino acid position of the predicted interface on Protein A; ProtB: identifier of the human protein; ProtB_interface_id: Identifier of the predicted interface (an InterPro domain); ProtB_interface_start: starting amino acid position of the predicted interface on Protein B; ProtB_interface_end: ending amino acid position of the predicted interface on ProteinB.

| **ProtA** | **ProtA_interface_id** | **ProtA_interface_start** | **ProtA_interface_end** | **Prot_B** | **ProtB_interface_id** | **ProtB_interface_start** | **ProtB_interface_end** |
| --- | --- | --- | --- | --- | --- | --- | --- |
| Q820B6 | IPR000719 | 26 | 174 | P23515 | IPR001611 | 79 | 100 |
| Q820B6 | IPR000719 | 26 | 174 | P23515 | IPR001611 | 147 | 168 |
| Q820B6 | IPR000719 | 26 | 174 | P23515 | IPR001611 | 168 | 226 |
| Q820B6 | IPR000719 | 26 | 174 | Q93062 | IPR000504 | 24 | 101 |
| Q83A11 | DEG_APCC_DBOX_1 | 16 | 24 | Q8TAF3 | IPR001680 | 14 | 60 |
| Q83A11 | DEG_APCC_DBOX_1 | 16 | 24 | Q8TAF3 | IPR001680 | 64 | 154 |
| Q83A11 | DEG_APCC_DBOX_1 | 16 | 24 | Q8TAF3 | IPR001680 | 157 | 280 |
| Q83A11 | DEG_APCC_DBOX_1 | 16 | 24 | Q8TAF3 | IPR001680 | 350 | 388 |
| Q83A11 | DEG_APCC_DBOX_1 | 16 | 24 | Q8TAF3 | IPR015943 | 24 | 157 |
| Q83A11 | DEG_APCC_DBOX_1 | 16 | 24 | Q8TAF3 | IPR015943 | 158 | 409 |
| Q83A11 | DEG_APCC_DBOX_1 | 16 | 24 | Q96EE3 | IPR001680 | 1 | 40 |
| Q83A11 | DEG_APCC_DBOX_1 | 16 | 24 | Q96EE3 | IPR001680 | 46 | 87 |
| Q83A11 | DEG_APCC_DBOX_1 | 16 | 24 | Q96EE3 | IPR001680 | 102 | 143 |
| Q83A11 | DEG_APCC_DBOX_1 | 16 | 24 | Q96EE3 | IPR001680 | 152 | 201 |
| Q83A11 | DEG_APCC_DBOX_1 | 16 | 24 | Q96EE3 | IPR001680 | 267 | 306 |
| Q83A11 | DEG_APCC_DBOX_1 | 16 | 24 | Q96EE3 | IPR015943 | 3 | 320 |
| Q83A11 | DEG_APCC_DBOX_1 | 16 | 24 | Q8TAF3 | IPR001680 | 14 | 60 |
| Q83A11 | DEG_APCC_DBOX_1 | 16 | 24 | Q8TAF3 | IPR001680 | 64 | 154 |
| Q83A11 | DEG_APCC_DBOX_1 | 16 | 24 | Q8TAF3 | IPR001680 | 157 | 280 |
| Q83A11 | DEG_APCC_DBOX_1 | 16 | 24 | Q8TAF3 | IPR001680 | 350 | 388 |
| Q83A11 | DEG_APCC_DBOX_1 | 16 | 24 | Q8TAF3 | IPR015943 | 24 | 157 |
| Q83A11 | DEG_APCC_DBOX_1 | 16 | 24 | Q8TAF3 | IPR015943 | 158 | 409 |
| Q83A11 | DOC_WW_Pin1_4 | 10 | 15 | Q8IX03 | IPR001202 | 6 | 39 |
| Q83A11 | DOC_WW_Pin1_4 | 10 | 15 | Q8IX03 | IPR001202 | 53 | 86 |
| Q83A33 | IPR003812 | 103 | 207 | P11021 | IPR013126 | 16 | 643 |
| Q83A33 | LIG_Integrin_RGD_1 | 121 | 123 | P17301 | IPR013517 | 491 | 530 |
| Q83A33 | LIG_Integrin_RGD_1 | 121 | 123 | P17301 | IPR013517 | 554 | 589 |
| Q83A33 | TRG_ER_diLys_1 | 343 | 348 | Q8TAF3 | IPR001680 | 14 | 60 |
| Q83A33 | TRG_ER_diLys_1 | 343 | 348 | Q8TAF3 | IPR001680 | 64 | 154 |
| Q83A33 | TRG_ER_diLys_1 | 343 | 348 | Q8TAF3 | IPR001680 | 157 | 280 |
| Q83A33 | TRG_ER_diLys_1 | 343 | 348 | Q8TAF3 | IPR001680 | 350 | 388 |
| Q83A33 | TRG_ER_diLys_1 | 343 | 348 | Q8TAF3 | IPR001680 | 14 | 60 |
| Q83A33 | TRG_ER_diLys_1 | 343 | 348 | Q8TAF3 | IPR001680 | 64 | 154 |
| Q83A33 | TRG_ER_diLys_1 | 343 | 348 | Q8TAF3 | IPR001680 | 157 | 280 |
| Q83A33 | TRG_ER_diLys_1 | 343 | 348 | Q8TAF3 | IPR001680 | 350 | 388 |
| Q83B01 | MOD_CK1_1 | 300 | 306 | Q9BWU1 | IPR000719 | 21 | 335 |
| Q83B01 | MOD_CK1_1 | 403 | 409 | Q9BWU1 | IPR000719 | 21 | 335 |
| Q83B01 | MOD_CK1_1 | 610 | 616 | Q9BWU1 | IPR000719 | 21 | 335 |
| Q83B01 | MOD_CK1_1 | 711 | 717 | Q9BWU1 | IPR000719 | 21 | 335 |
| Q83B01 | MOD_CK1_1 | 715 | 721 | Q9BWU1 | IPR000719 | 21 | 335 |
| Q83B01 | MOD_CK1_1 | 718 | 724 | Q9BWU1 | IPR000719 | 21 | 335 |
| Q83B01 | MOD_CK1_1 | 300 | 306 | P53779 | IPR000719 | 64 | 359 |
| Q83B01 | MOD_CK1_1 | 403 | 409 | P53779 | IPR000719 | 64 | 359 |
| Q83B01 | MOD_CK1_1 | 610 | 616 | P53779 | IPR000719 | 64 | 359 |
| Q83B01 | MOD_CK1_1 | 711 | 717 | P53779 | IPR000719 | 64 | 359 |
| Q83B01 | MOD_CK1_1 | 715 | 721 | P53779 | IPR000719 | 64 | 359 |
| Q83B01 | MOD_CK1_1 | 718 | 724 | P53779 | IPR000719 | 64 | 359 |
| Q83B01 | MOD_CK1_1 | 300 | 306 | P53779 | IPR000719 | 64 | 359 |
| Q83B01 | MOD_CK1_1 | 403 | 409 | P53779 | IPR000719 | 64 | 359 |
| Q83B01 | MOD_CK1_1 | 610 | 616 | P53779 | IPR000719 | 64 | 359 |
| Q83B01 | MOD_CK1_1 | 711 | 717 | P53779 | IPR000719 | 64 | 359 |
| Q83B01 | MOD_CK1_1 | 715 | 721 | P53779 | IPR000719 | 64 | 359 |
| Q83B01 | MOD_CK1_1 | 718 | 724 | P53779 | IPR000719 | 64 | 359 |
| Q83B01 | MOD_CK2_1 | 300 | 306 | Q9BWU1 | IPR000719 | 21 | 335 |
| Q83B01 | MOD_CK2_1 | 403 | 409 | Q9BWU1 | IPR000719 | 21 | 335 |
| Q83B01 | MOD_CK2_1 | 404 | 410 | Q9BWU1 | IPR000719 | 21 | 335 |
| Q83B01 | MOD_CK2_1 | 642 | 648 | Q9BWU1 | IPR000719 | 21 | 335 |
| Q83B01 | MOD_CK2_1 | 416 | 422 | Q9BWU1 | IPR000719 | 21 | 335 |
| Q83B01 | MOD_CK2_1 | 416 | 422 | P53779 | IPR000719 | 64 | 359 |
| Q83B01 | MOD_CK2_1 | 300 | 306 | P53779 | IPR000719 | 64 | 359 |
| Q83B01 | MOD_CK2_1 | 403 | 409 | P53779 | IPR000719 | 64 | 359 |
| Q83B01 | MOD_CK2_1 | 404 | 410 | P53779 | IPR000719 | 64 | 359 |
| Q83B01 | MOD_CK2_1 | 642 | 648 | P53779 | IPR000719 | 64 | 359 |
| Q83B01 | MOD_CK2_1 | 416 | 422 | P53779 | IPR000719 | 64 | 359 |
| Q83B01 | MOD_CK2_1 | 300 | 306 | P53779 | IPR000719 | 64 | 359 |
| Q83B01 | MOD_CK2_1 | 403 | 409 | P53779 | IPR000719 | 64 | 359 |
| Q83B01 | MOD_CK2_1 | 404 | 410 | P53779 | IPR000719 | 64 | 359 |
| Q83B01 | MOD_CK2_1 | 642 | 648 | P53779 | IPR000719 | 64 | 359 |
| Q83B01 | MOD_GSK3_1 | 356 | 363 | Q9BWU1 | IPR000719 | 21 | 335 |
| Q83B01 | MOD_GSK3_1 | 400 | 407 | Q9BWU1 | IPR000719 | 21 | 335 |
| Q83B01 | MOD_GSK3_1 | 404 | 411 | Q9BWU1 | IPR000719 | 21 | 335 |
| Q83B01 | MOD_GSK3_1 | 607 | 614 | Q9BWU1 | IPR000719 | 21 | 335 |
| Q83B01 | MOD_GSK3_1 | 708 | 715 | Q9BWU1 | IPR000719 | 21 | 335 |
| Q83B01 | MOD_GSK3_1 | 711 | 718 | Q9BWU1 | IPR000719 | 21 | 335 |
| Q83B01 | MOD_GSK3_1 | 715 | 722 | Q9BWU1 | IPR000719 | 21 | 335 |
| Q83B01 | MOD_GSK3_1 | 356 | 363 | P53779 | IPR000719 | 64 | 359 |
| Q83B01 | MOD_GSK3_1 | 400 | 407 | P53779 | IPR000719 | 64 | 359 |
| Q83B01 | MOD_GSK3_1 | 404 | 411 | P53779 | IPR000719 | 64 | 359 |
| Q83B01 | MOD_GSK3_1 | 607 | 614 | P53779 | IPR000719 | 64 | 359 |
| Q83B01 | MOD_GSK3_1 | 708 | 715 | P53779 | IPR000719 | 64 | 359 |
| Q83B01 | MOD_GSK3_1 | 711 | 718 | P53779 | IPR000719 | 64 | 359 |
| Q83B01 | MOD_GSK3_1 | 715 | 722 | P53779 | IPR000719 | 64 | 359 |
| Q83B01 | MOD_GSK3_1 | 356 | 363 | P53779 | IPR000719 | 64 | 359 |
| Q83B01 | MOD_GSK3_1 | 400 | 407 | P53779 | IPR000719 | 64 | 359 |
| Q83B01 | MOD_GSK3_1 | 404 | 411 | P53779 | IPR000719 | 64 | 359 |
| Q83B01 | MOD_GSK3_1 | 607 | 614 | P53779 | IPR000719 | 64 | 359 |
| Q83B01 | MOD_GSK3_1 | 708 | 715 | P53779 | IPR000719 | 64 | 359 |
| Q83B01 | MOD_GSK3_1 | 711 | 718 | P53779 | IPR000719 | 64 | 359 |
| Q83B01 | MOD_GSK3_1 | 715 | 722 | P53779 | IPR000719 | 64 | 359 |
| Q83B01 | MOD_PKA_2 | 297 | 303 | Q9BWU1 | IPR000719 | 21 | 335 |
| Q83B01 | MOD_PKA_2 | 607 | 613 | Q9BWU1 | IPR000719 | 21 | 335 |
| Q83B01 | MOD_PKA_2 | 297 | 303 | P53779 | IPR000719 | 64 | 359 |
| Q83B01 | MOD_PKA_2 | 607 | 613 | P53779 | IPR000719 | 64 | 359 |
| Q83B01 | MOD_PKA_2 | 297 | 303 | P53779 | IPR000719 | 64 | 359 |
| Q83B01 | MOD_PKA_2 | 607 | 613 | P53779 | IPR000719 | 64 | 359 |
| Q83B01 | MOD_Plk_1 | 610 | 616 | Q9BWU1 | IPR000719 | 21 | 335 |
| Q83B01 | MOD_Plk_1 | 610 | 616 | P53779 | IPR000719 | 64 | 359 |
| Q83B01 | MOD_Plk_1 | 610 | 616 | P53779 | IPR000719 | 64 | 359 |
| Q83B01 | MOD_Plk_2-3 | 274 | 280 | Q9BWU1 | IPR000719 | 21 | 335 |
| Q83B01 | MOD_Plk_2-3 | 611 | 617 | Q9BWU1 | IPR000719 | 21 | 335 |
| Q83B01 | MOD_Plk_2-3 | 274 | 280 | P53779 | IPR000719 | 64 | 359 |
| Q83B01 | MOD_Plk_2-3 | 611 | 617 | P53779 | IPR000719 | 64 | 359 |
| Q83B01 | MOD_Plk_2-3 | 274 | 280 | P53779 | IPR000719 | 64 | 359 |
| Q83B01 | MOD_Plk_2-3 | 611 | 617 | P53779 | IPR000719 | 64 | 359 |
| Q83B01 | MOD_Plk_4 | 410 | 416 | Q9BWU1 | IPR000719 | 21 | 335 |
| Q83B01 | MOD_Plk_4 | 560 | 566 | Q9BWU1 | IPR000719 | 21 | 335 |
| Q83B01 | MOD_Plk_4 | 286 | 292 | Q9BWU1 | IPR000719 | 21 | 335 |
| Q83B01 | MOD_Plk_4 | 286 | 292 | P53779 | IPR000719 | 64 | 359 |
| Q83B01 | MOD_Plk_4 | 410 | 416 | P53779 | IPR000719 | 64 | 359 |
| Q83B01 | MOD_Plk_4 | 560 | 566 | P53779 | IPR000719 | 64 | 359 |
| Q83B01 | MOD_Plk_4 | 286 | 292 | P53779 | IPR000719 | 64 | 359 |
| Q83B01 | MOD_Plk_4 | 410 | 416 | P53779 | IPR000719 | 64 | 359 |
| Q83B01 | MOD_Plk_4 | 560 | 566 | P53779 | IPR000719 | 64 | 359 |
| Q83B01 | MOD_ProDKin_1 | 401 | 407 | Q9BWU1 | IPR000719 | 21 | 335 |
| Q83B01 | MOD_ProDKin_1 | 708 | 714 | Q9BWU1 | IPR000719 | 21 | 335 |
| Q83B01 | MOD_ProDKin_1 | 472 | 478 | Q9BWU1 | IPR000719 | 21 | 335 |
| Q83B01 | MOD_ProDKin_1 | 472 | 478 | P53779 | IPR000719 | 64 | 359 |
| Q83B01 | MOD_ProDKin_1 | 401 | 407 | P53779 | IPR000719 | 64 | 359 |
| Q83B01 | MOD_ProDKin_1 | 708 | 714 | P53779 | IPR000719 | 64 | 359 |
| Q83B01 | MOD_ProDKin_1 | 472 | 478 | P53779 | IPR000719 | 64 | 359 |
| Q83B01 | MOD_ProDKin_1 | 401 | 407 | P53779 | IPR000719 | 64 | 359 |
| Q83B01 | MOD_ProDKin_1 | 708 | 714 | P53779 | IPR000719 | 64 | 359 |
| Q83D09 | DOC_MAPK_gen_1 | 237 | 245 | P45985 | IPR000719 | 102 | 367 |
| Q83D09 | DOC_PP2B_LxvP_1 | 121 | 124 | Q9Y680 | IPR002048 | 189 | 222 |
| Q83D09 | LIG_PDZ_Class_2 | 460 | 465 | Q5T2W1 | IPR001478 | 9 | 90 |
| Q83D09 | LIG_PDZ_Class_2 | 460 | 465 | Q5T2W1 | IPR001478 | 134 | 215 |
| Q83D09 | LIG_PDZ_Class_2 | 460 | 465 | Q5T2W1 | IPR001478 | 243 | 323 |
| Q83D09 | LIG_PDZ_Class_2 | 460 | 465 | Q5T2W1 | IPR001478 | 378 | 458 |
| Q83D09 | MOD_CDK_SPxK_1 | 439 | 445 | P45985 | IPR000719 | 102 | 367 |
| Q83D09 | MOD_CK1_1 | 66 | 72 | P45985 | IPR000719 | 102 | 367 |
| Q83D09 | MOD_CK1_1 | 75 | 81 | P45985 | IPR000719 | 102 | 367 |
| Q83D09 | MOD_CK1_1 | 142 | 148 | P45985 | IPR000719 | 102 | 367 |
| Q83D09 | MOD_CK1_1 | 299 | 305 | P45985 | IPR000719 | 102 | 367 |
| Q83D09 | MOD_CK1_1 | 381 | 387 | P45985 | IPR000719 | 102 | 367 |
| Q83D09 | MOD_CK1_1 | 436 | 442 | P45985 | IPR000719 | 102 | 367 |
| Q83D09 | MOD_CK2_1 | 155 | 161 | P45985 | IPR000719 | 102 | 367 |
| Q83D09 | MOD_GSK3_1 | 17 | 24 | P45985 | IPR000719 | 102 | 367 |
| Q83D09 | MOD_GSK3_1 | 18 | 25 | P45985 | IPR000719 | 102 | 367 |
| Q83D09 | MOD_GSK3_1 | 21 | 28 | P45985 | IPR000719 | 102 | 367 |
| Q83D09 | MOD_GSK3_1 | 22 | 29 | P45985 | IPR000719 | 102 | 367 |
| Q83D09 | MOD_GSK3_1 | 71 | 78 | P45985 | IPR000719 | 102 | 367 |
| Q83D09 | MOD_GSK3_1 | 138 | 145 | P45985 | IPR000719 | 102 | 367 |
| Q83D09 | MOD_GSK3_1 | 139 | 146 | P45985 | IPR000719 | 102 | 367 |
| Q83D09 | MOD_GSK3_1 | 143 | 150 | P45985 | IPR000719 | 102 | 367 |
| Q83D09 | MOD_GSK3_1 | 225 | 232 | P45985 | IPR000719 | 102 | 367 |
| Q83D09 | MOD_GSK3_1 | 263 | 270 | P45985 | IPR000719 | 102 | 367 |
| Q83D09 | MOD_GSK3_1 | 364 | 371 | P45985 | IPR000719 | 102 | 367 |
| Q83D09 | MOD_GSK3_1 | 378 | 385 | P45985 | IPR000719 | 102 | 367 |
| Q83D09 | MOD_PKA_1 | 63 | 69 | P45985 | IPR000719 | 102 | 367 |
| Q83D09 | MOD_PKA_2 | 290 | 296 | P45985 | IPR000719 | 102 | 367 |
| Q83D09 | MOD_PKA_2 | 75 | 81 | P45985 | IPR000719 | 102 | 367 |
| Q83D09 | MOD_PKA_2 | 364 | 370 | P45985 | IPR000719 | 102 | 367 |
| Q83D09 | MOD_PKB_1 | 239 | 247 | P45985 | IPR000719 | 102 | 367 |
| Q83D09 | MOD_Plk_1 | 373 | 379 | P45985 | IPR000719 | 102 | 367 |
| Q83D09 | MOD_Plk_4 | 296 | 302 | P45985 | IPR000719 | 102 | 367 |
| Q83D09 | MOD_ProDKin_1 | 22 | 28 | P45985 | IPR000719 | 102 | 367 |
| Q83D09 | MOD_ProDKin_1 | 226 | 232 | P45985 | IPR000719 | 102 | 367 |
| Q83D09 | MOD_ProDKin_1 | 439 | 445 | P45985 | IPR000719 | 102 | 367 |
| Q83D60 | LIG_BRCT_BRCA1_1 | 4 | 8 | Q92547 | IPR001357 | 101 | 179 |
| Q83D60 | LIG_BRCT_BRCA1_1 | 4 | 8 | Q92547 | IPR001357 | 195 | 284 |
| Q83D60 | LIG_BRCT_BRCA1_1 | 4 | 8 | Q92547 | IPR001357 | 354 | 444 |
| Q83D60 | LIG_BRCT_BRCA1_1 | 4 | 8 | Q92547 | IPR001357 | 548 | 633 |
| Q83D60 | LIG_BRCT_BRCA1_1 | 4 | 8 | Q92547 | IPR001357 | 641 | 738 |
| Q83D60 | LIG_BRCT_BRCA1_1 | 4 | 8 | Q92547 | IPR001357 | 900 | 991 |
| Q83D60 | LIG_BRCT_BRCA1_1 | 4 | 8 | Q92547 | IPR001357 | 1259 | 1351 |
| Q83D60 | LIG_NRP_CendR_1 | 218 | 221 | P12259 | IPR000421 | 1906 | 2061 |
| Q83D60 | LIG_NRP_CendR_1 | 218 | 221 | P12259 | IPR000421 | 2065 | 2221 |
| Q83D60 | MOD_CK2_1 | 7 | 13 | P51955 | IPR000719 | 8 | 271 |
| Q83D60 | MOD_CK2_1 | 7 | 13 | P51955 | IPR000719 | 8 | 271 |
| Q83D60 | MOD_GSK3_1 | 4 | 11 | P51955 | IPR000719 | 8 | 271 |
| Q83D60 | MOD_GSK3_1 | 4 | 11 | P51955 | IPR000719 | 8 | 271 |
| Q83D60 | MOD_Plk_4 | 4 | 10 | P51955 | IPR000719 | 8 | 271 |
| Q83D60 | MOD_Plk_4 | 4 | 10 | P51955 | IPR000719 | 8 | 271 |
| Q83D60 | MOD_ProDKin_1 | 2 | 8 | P51955 | IPR000719 | 8 | 271 |
| Q83D60 | MOD_ProDKin_1 | 2 | 8 | P51955 | IPR000719 | 8 | 271 |
| Q83DE4 | DOC_PP2A_B56_1 | 237 | 242 | Q96PY5 | IPR016024 | 31 | 451 |
| Q83DE4 | LIG_NRP_CendR_1 | 462 | 464 | O14786 | IPR000421 | 274 | 424 |
| Q83DE4 | LIG_NRP_CendR_1 | 462 | 464 | O14786 | IPR000421 | 430 | 583 |
| Q83DF6 | DOC_MAPK_gen_1 | 231 | 240 | O94921 | IPR000719 | 135 | 419 |
| Q83DF6 | DOC_MAPK_gen_1 | 233 | 240 | O94921 | IPR000719 | 135 | 419 |
| Q83DF6 | DOC_MAPK_gen_1 | 231 | 240 | O15075 | IPR000719 | 390 | 647 |
| Q83DF6 | DOC_MAPK_gen_1 | 233 | 240 | O15075 | IPR000719 | 390 | 647 |
| Q83DF6 | DOC_PP2A_B56_1 | 305 | 310 | O00505 | IPR016024 | 9 | 487 |
| Q83DF6 | MOD_CDK_SPK_2 | 17 | 22 | O94921 | IPR000719 | 135 | 419 |
| Q83DF6 | MOD_CDK_SPK_2 | 333 | 338 | O94921 | IPR000719 | 135 | 419 |
| Q83DF6 | MOD_CDK_SPK_2 | 17 | 22 | O15075 | IPR000719 | 390 | 647 |
| Q83DF6 | MOD_CDK_SPK_2 | 333 | 338 | O15075 | IPR000719 | 390 | 647 |
| Q83DF6 | MOD_CDK_SPxK_1 | 17 | 23 | O94921 | IPR000719 | 135 | 419 |
| Q83DF6 | MOD_CDK_SPxK_1 | 17 | 23 | O15075 | IPR000719 | 390 | 647 |
| Q83DF6 | MOD_CDK_SPxxK_3 | 17 | 24 | O94921 | IPR000719 | 135 | 419 |
| Q83DF6 | MOD_CDK_SPxxK_3 | 17 | 24 | O15075 | IPR000719 | 390 | 647 |
| Q83DF6 | MOD_CK1_1 | 9 | 15 | O94921 | IPR000719 | 135 | 419 |
| Q83DF6 | MOD_CK1_1 | 13 | 19 | O94921 | IPR000719 | 135 | 419 |
| Q83DF6 | MOD_CK1_1 | 194 | 200 | O94921 | IPR000719 | 135 | 419 |
| Q83DF6 | MOD_CK1_1 | 332 | 338 | O94921 | IPR000719 | 135 | 419 |
| Q83DF6 | MOD_CK1_1 | 9 | 15 | O15075 | IPR000719 | 390 | 647 |
| Q83DF6 | MOD_CK1_1 | 13 | 19 | O15075 | IPR000719 | 390 | 647 |
| Q83DF6 | MOD_CK1_1 | 194 | 200 | O15075 | IPR000719 | 390 | 647 |
| Q83DF6 | MOD_CK1_1 | 332 | 338 | O15075 | IPR000719 | 390 | 647 |
| Q83DF6 | MOD_CK2_1 | 199 | 205 | O94921 | IPR000719 | 135 | 419 |
| Q83DF6 | MOD_CK2_1 | 280 | 286 | O94921 | IPR000719 | 135 | 419 |
| Q83DF6 | MOD_CK2_1 | 199 | 205 | O15075 | IPR000719 | 390 | 647 |
| Q83DF6 | MOD_CK2_1 | 280 | 286 | O15075 | IPR000719 | 390 | 647 |
| Q83DF6 | MOD_GSK3_1 | 3 | 10 | O94921 | IPR000719 | 135 | 419 |
| Q83DF6 | MOD_GSK3_1 | 5 | 12 | O94921 | IPR000719 | 135 | 419 |
| Q83DF6 | MOD_GSK3_1 | 6 | 13 | O94921 | IPR000719 | 135 | 419 |
| Q83DF6 | MOD_GSK3_1 | 7 | 14 | O94921 | IPR000719 | 135 | 419 |
| Q83DF6 | MOD_GSK3_1 | 9 | 16 | O94921 | IPR000719 | 135 | 419 |
| Q83DF6 | MOD_GSK3_1 | 11 | 18 | O94921 | IPR000719 | 135 | 419 |
| Q83DF6 | MOD_GSK3_1 | 13 | 20 | O94921 | IPR000719 | 135 | 419 |
| Q83DF6 | MOD_GSK3_1 | 232 | 239 | O94921 | IPR000719 | 135 | 419 |
| Q83DF6 | MOD_GSK3_1 | 323 | 330 | O94921 | IPR000719 | 135 | 419 |
| Q83DF6 | MOD_GSK3_1 | 329 | 336 | O94921 | IPR000719 | 135 | 419 |
| Q83DF6 | MOD_GSK3_1 | 3 | 10 | O15075 | IPR000719 | 390 | 647 |
| Q83DF6 | MOD_GSK3_1 | 5 | 12 | O15075 | IPR000719 | 390 | 647 |
| Q83DF6 | MOD_GSK3_1 | 6 | 13 | O15075 | IPR000719 | 390 | 647 |
| Q83DF6 | MOD_GSK3_1 | 7 | 14 | O15075 | IPR000719 | 390 | 647 |
| Q83DF6 | MOD_GSK3_1 | 9 | 16 | O15075 | IPR000719 | 390 | 647 |
| Q83DF6 | MOD_GSK3_1 | 11 | 18 | O15075 | IPR000719 | 390 | 647 |
| Q83DF6 | MOD_GSK3_1 | 13 | 20 | O15075 | IPR000719 | 390 | 647 |
| Q83DF6 | MOD_GSK3_1 | 232 | 239 | O15075 | IPR000719 | 390 | 647 |
| Q83DF6 | MOD_GSK3_1 | 323 | 330 | O15075 | IPR000719 | 390 | 647 |
| Q83DF6 | MOD_GSK3_1 | 329 | 336 | O15075 | IPR000719 | 390 | 647 |
| Q83DF6 | MOD_PKA_1 | 23 | 29 | O94921 | IPR000719 | 135 | 419 |
| Q83DF6 | MOD_PKA_1 | 23 | 29 | O15075 | IPR000719 | 390 | 647 |
| Q83DF6 | MOD_PKA_2 | 23 | 29 | O94921 | IPR000719 | 135 | 419 |
| Q83DF6 | MOD_PKA_2 | 23 | 29 | O15075 | IPR000719 | 390 | 647 |
| Q83DF6 | MOD_Plk_1 | 162 | 168 | O94921 | IPR000719 | 135 | 419 |
| Q83DF6 | MOD_Plk_1 | 292 | 298 | O94921 | IPR000719 | 135 | 419 |
| Q83DF6 | MOD_Plk_1 | 320 | 326 | O94921 | IPR000719 | 135 | 419 |
| Q83DF6 | MOD_Plk_1 | 321 | 327 | O94921 | IPR000719 | 135 | 419 |
| Q83DF6 | MOD_Plk_1 | 327 | 333 | O94921 | IPR000719 | 135 | 419 |
| Q83DF6 | MOD_Plk_1 | 162 | 168 | O15075 | IPR000719 | 390 | 647 |
| Q83DF6 | MOD_Plk_1 | 292 | 298 | O15075 | IPR000719 | 390 | 647 |
| Q83DF6 | MOD_Plk_1 | 320 | 326 | O15075 | IPR000719 | 390 | 647 |
| Q83DF6 | MOD_Plk_1 | 321 | 327 | O15075 | IPR000719 | 390 | 647 |
| Q83DF6 | MOD_Plk_1 | 327 | 333 | O15075 | IPR000719 | 390 | 647 |
| Q83DF6 | MOD_Plk_4 | 92 | 98 | O94921 | IPR000719 | 135 | 419 |
| Q83DF6 | MOD_Plk_4 | 92 | 98 | O15075 | IPR000719 | 390 | 647 |
| Q83DF6 | MOD_ProDKin_1 | 3 | 9 | O94921 | IPR000719 | 135 | 419 |
| Q83DF6 | MOD_ProDKin_1 | 11 | 17 | O94921 | IPR000719 | 135 | 419 |
| Q83DF6 | MOD_ProDKin_1 | 17 | 23 | O94921 | IPR000719 | 135 | 419 |
| Q83DF6 | MOD_ProDKin_1 | 32 | 38 | O94921 | IPR000719 | 135 | 419 |
| Q83DF6 | MOD_ProDKin_1 | 3 | 9 | O15075 | IPR000719 | 390 | 647 |
| Q83DF6 | MOD_ProDKin_1 | 11 | 17 | O15075 | IPR000719 | 390 | 647 |
| Q83DF6 | MOD_ProDKin_1 | 17 | 23 | O15075 | IPR000719 | 390 | 647 |
| Q83DF6 | MOD_ProDKin_1 | 32 | 38 | O15075 | IPR000719 | 390 | 647 |
| Q83DF6 | TRG_NLS_MonoExtN_4 | 231 | 237 | O00505 | IPR000225 | 103 | 186 |
| Q83DF6 | TRG_NLS_MonoExtN_4 | 231 | 237 | O00505 | IPR000225 | 188 | 229 |
| Q83DF6 | TRG_NLS_MonoExtN_4 | 231 | 237 | O00505 | IPR000225 | 232 | 271 |
| Q83DF6 | TRG_NLS_MonoExtN_4 | 231 | 237 | O00505 | IPR000225 | 273 | 355 |
| Q83DF6 | TRG_NLS_MonoExtN_4 | 231 | 237 | O00505 | IPR000225 | 357 | 397 |
| Q83DF6 | TRG_NLS_MonoExtN_4 | 231 | 237 | O00505 | IPR000225 | 400 | 440 |
| Q83FA5 | DEG_APCC_DBOX_1 | 337 | 345 | Q9NZJ5 | IPR015943 | 93 | 397 |
| Q83FA5 | DOC_MAPK_gen_1 | 14 | 23 | Q12866 | IPR000719 | 587 | 858 |
| Q83FA5 | DOC_MAPK_gen_1 | 335 | 343 | Q12866 | IPR000719 | 587 | 858 |
| Q83FA5 | DOC_MAPK_gen_1 | 336 | 343 | Q12866 | IPR000719 | 587 | 858 |
| Q83FA5 | DOC_MAPK_gen_1 | 14 | 23 | Q9NZJ5 | IPR000719 | 593 | 1077 |
| Q83FA5 | DOC_MAPK_gen_1 | 335 | 343 | Q9NZJ5 | IPR000719 | 593 | 1077 |
| Q83FA5 | DOC_MAPK_gen_1 | 336 | 343 | Q9NZJ5 | IPR000719 | 593 | 1077 |
| Q83FA5 | MOD_CDK_SPxxK_3 | 621 | 628 | Q12866 | IPR000719 | 587 | 858 |
| Q83FA5 | MOD_CDK_SPxxK_3 | 621 | 628 | Q9NZJ5 | IPR000719 | 593 | 1077 |
| Q83FA5 | MOD_CK1_1 | 198 | 204 | Q12866 | IPR000719 | 587 | 858 |
| Q83FA5 | MOD_CK1_1 | 219 | 225 | Q12866 | IPR000719 | 587 | 858 |
| Q83FA5 | MOD_CK1_1 | 545 | 551 | Q12866 | IPR000719 | 587 | 858 |
| Q83FA5 | MOD_CK1_1 | 579 | 585 | Q12866 | IPR000719 | 587 | 858 |
| Q83FA5 | MOD_CK1_1 | 593 | 599 | Q12866 | IPR000719 | 587 | 858 |
| Q83FA5 | MOD_CK1_1 | 681 | 687 | Q12866 | IPR000719 | 587 | 858 |
| Q83FA5 | MOD_CK1_1 | 684 | 690 | Q12866 | IPR000719 | 587 | 858 |
| Q83FA5 | MOD_CK1_1 | 198 | 204 | Q9NZJ5 | IPR000719 | 593 | 1077 |
| Q83FA5 | MOD_CK1_1 | 219 | 225 | Q9NZJ5 | IPR000719 | 593 | 1077 |
| Q83FA5 | MOD_CK1_1 | 545 | 551 | Q9NZJ5 | IPR000719 | 593 | 1077 |
| Q83FA5 | MOD_CK1_1 | 579 | 585 | Q9NZJ5 | IPR000719 | 593 | 1077 |
| Q83FA5 | MOD_CK1_1 | 593 | 599 | Q9NZJ5 | IPR000719 | 593 | 1077 |
| Q83FA5 | MOD_CK1_1 | 681 | 687 | Q9NZJ5 | IPR000719 | 593 | 1077 |
| Q83FA5 | MOD_CK1_1 | 684 | 690 | Q9NZJ5 | IPR000719 | 593 | 1077 |
| Q83FA5 | MOD_CK2_1 | 198 | 204 | Q12866 | IPR000719 | 587 | 858 |
| Q83FA5 | MOD_CK2_1 | 336 | 342 | Q12866 | IPR000719 | 587 | 858 |
| Q83FA5 | MOD_CK2_1 | 198 | 204 | Q9NZJ5 | IPR000719 | 593 | 1077 |
| Q83FA5 | MOD_CK2_1 | 336 | 342 | Q9NZJ5 | IPR000719 | 593 | 1077 |
| Q83FA5 | MOD_GSK3_1 | 3 | 10 | Q12866 | IPR000719 | 587 | 858 |
| Q83FA5 | MOD_GSK3_1 | 31 | 38 | Q12866 | IPR000719 | 587 | 858 |
| Q83FA5 | MOD_GSK3_1 | 545 | 552 | Q12866 | IPR000719 | 587 | 858 |
| Q83FA5 | MOD_GSK3_1 | 576 | 583 | Q12866 | IPR000719 | 587 | 858 |
| Q83FA5 | MOD_GSK3_1 | 628 | 635 | Q12866 | IPR000719 | 587 | 858 |
| Q83FA5 | MOD_GSK3_1 | 632 | 639 | Q12866 | IPR000719 | 587 | 858 |
| Q83FA5 | MOD_GSK3_1 | 671 | 678 | Q12866 | IPR000719 | 587 | 858 |
| Q83FA5 | MOD_GSK3_1 | 678 | 685 | Q12866 | IPR000719 | 587 | 858 |
| Q83FA5 | MOD_GSK3_1 | 694 | 701 | Q12866 | IPR000719 | 587 | 858 |
| Q83FA5 | MOD_GSK3_1 | 3 | 10 | Q9NZJ5 | IPR000719 | 593 | 1077 |
| Q83FA5 | MOD_GSK3_1 | 31 | 38 | Q9NZJ5 | IPR000719 | 593 | 1077 |
| Q83FA5 | MOD_GSK3_1 | 545 | 552 | Q9NZJ5 | IPR000719 | 593 | 1077 |
| Q83FA5 | MOD_GSK3_1 | 576 | 583 | Q9NZJ5 | IPR000719 | 593 | 1077 |
| Q83FA5 | MOD_GSK3_1 | 628 | 635 | Q9NZJ5 | IPR000719 | 593 | 1077 |
| Q83FA5 | MOD_GSK3_1 | 632 | 639 | Q9NZJ5 | IPR000719 | 593 | 1077 |
| Q83FA5 | MOD_GSK3_1 | 671 | 678 | Q9NZJ5 | IPR000719 | 593 | 1077 |
| Q83FA5 | MOD_GSK3_1 | 678 | 685 | Q9NZJ5 | IPR000719 | 593 | 1077 |
| Q83FA5 | MOD_GSK3_1 | 694 | 701 | Q9NZJ5 | IPR000719 | 593 | 1077 |
| Q83FA5 | MOD_LATS_1 | 626 | 632 | Q12866 | IPR000719 | 587 | 858 |
| Q83FA5 | MOD_LATS_1 | 626 | 632 | Q9NZJ5 | IPR000719 | 593 | 1077 |
| Q83FA5 | MOD_PKA_2 | 413 | 419 | Q12866 | IPR000719 | 587 | 858 |
| Q83FA5 | MOD_PKA_2 | 192 | 198 | Q12866 | IPR000719 | 587 | 858 |
| Q83FA5 | MOD_PKA_2 | 418 | 424 | Q12866 | IPR000719 | 587 | 858 |
| Q83FA5 | MOD_PKA_2 | 604 | 610 | Q12866 | IPR000719 | 587 | 858 |
| Q83FA5 | MOD_PKA_2 | 192 | 198 | Q9NZJ5 | IPR000719 | 593 | 1077 |
| Q83FA5 | MOD_PKA_2 | 418 | 424 | Q9NZJ5 | IPR000719 | 593 | 1077 |
| Q83FA5 | MOD_PKA_2 | 604 | 610 | Q9NZJ5 | IPR000719 | 593 | 1077 |
| Q83FA5 | MOD_PKA_2 | 413 | 419 | Q9NZJ5 | IPR000719 | 593 | 1077 |
| Q83FA5 | MOD_Plk_1 | 593 | 599 | Q12866 | IPR000719 | 587 | 858 |
| Q83FA5 | MOD_Plk_1 | 593 | 599 | Q9NZJ5 | IPR000719 | 593 | 1077 |
| Q83FA5 | MOD_Plk_4 | 413 | 419 | Q12866 | IPR000719 | 587 | 858 |
| Q83FA5 | MOD_Plk_4 | 374 | 380 | Q12866 | IPR000719 | 587 | 858 |
| Q83FA5 | MOD_Plk_4 | 678 | 684 | Q12866 | IPR000719 | 587 | 858 |
| Q83FA5 | MOD_Plk_4 | 684 | 690 | Q12866 | IPR000719 | 587 | 858 |
| Q83FA5 | MOD_Plk_4 | 374 | 380 | Q9NZJ5 | IPR000719 | 593 | 1077 |
| Q83FA5 | MOD_Plk_4 | 678 | 684 | Q9NZJ5 | IPR000719 | 593 | 1077 |
| Q83FA5 | MOD_Plk_4 | 684 | 690 | Q9NZJ5 | IPR000719 | 593 | 1077 |
| Q83FA5 | MOD_Plk_4 | 413 | 419 | Q9NZJ5 | IPR000719 | 593 | 1077 |
| Q83FA5 | MOD_ProDKin_1 | 621 | 627 | Q12866 | IPR000719 | 587 | 858 |
| Q83FA5 | MOD_ProDKin_1 | 694 | 700 | Q12866 | IPR000719 | 587 | 858 |
| Q83FA5 | MOD_ProDKin_1 | 621 | 627 | Q9NZJ5 | IPR000719 | 593 | 1077 |
| Q83FA5 | MOD_ProDKin_1 | 694 | 700 | Q9NZJ5 | IPR000719 | 593 | 1077 |
